# Optineurin: A Coordinator of Membrane-Associated Cargo Trafficking and Autophagy

**DOI:** 10.3389/fimmu.2018.01024

**Published:** 2018-05-15

**Authors:** Thomas A. Ryan, David A. Tumbarello

**Affiliations:** Biological Sciences, University of Southampton, Southampton, United Kingdom

**Keywords:** amyotrophic lateral sclerosis, autophagy, cell signalling, glaucoma, Golgi, membrane trafficking, mitophagy, xenophagy

## Abstract

Optineurin is a multifunctional adaptor protein intimately involved in various vesicular trafficking pathways. Through interactions with an array of proteins, such as myosin VI, huntingtin, Rab8, and Tank-binding kinase 1, as well as via its oligomerisation, optineurin has the ability to act as an adaptor, scaffold, or signal regulator to coordinate many cellular processes associated with the trafficking of membrane-delivered cargo. Due to its diverse interactions and its distinct functions, optineurin is an essential component in a number of homeostatic pathways, such as protein trafficking and organelle maintenance. Through the binding of polyubiquitinated cargoes via its ubiquitin-binding domain, optineurin also serves as a selective autophagic receptor for the removal of a wide range of substrates. Alternatively, it can act in an ubiquitin-independent manner to mediate the clearance of protein aggregates. Regarding its disease associations, mutations in the optineurin gene are associated with glaucoma and have more recently been found to correlate with Paget’s disease of bone and amyotrophic lateral sclerosis (ALS). Indeed, ALS-associated mutations in optineurin result in defects in neuronal vesicular localisation, autophagosome–lysosome fusion, and secretory pathway function. More recent molecular and functional analysis has shown that it also plays a role in mitophagy, thus linking it to a number of other neurodegenerative conditions, such as Parkinson’s. Here, we review the role of optineurin in intracellular membrane trafficking, with a focus on autophagy, and describe how upstream signalling cascades are critical to its regulation. Current data and contradicting reports would suggest that optineurin is an important and selective autophagy receptor under specific conditions, whereby interplay, synergy, and functional redundancy with other receptors occurs. We will also discuss how dysfunction in optineurin-mediated pathways may lead to perturbation of critical cellular processes, which can drive the pathologies of number of diseases. Therefore, further understanding of optineurin function, its target specificity, and its mechanism of action will be critical in fully delineating its role in human disease.

## Introduction

Optineurin, through a diverse set of interactions, regulates a number of crucial cellular processes, specifically those that require the coordinated trafficking of protein and membrane cargo. First isolated in 1998 in a yeast two-hybrid screen by its interaction with the adenoviral protein E3-14.7K, it was initially named 14.7K-interacting protein (FIP-2) (1). A later study identified that mutations in this gene, located on chromosome 10p14, were found to associate with normal tension glaucoma (NTG), a subtype of primary open-angle glaucoma (POAG) (2). Thus, it was designated *OPTN*, encoding the optineurin (for “optic neuropathy inducing”) protein.

Since then, optineurin has been implicated as a genetic risk factor in Paget’s disease of bone (3, 4), familial and sporadic forms of amyotrophic lateral sclerosis (ALS) (5–11) and Crohn’s disease (12). In addition, optineurin has also been found to localise to an array of intracellular structures and compartments, providing evidence of its ubiquitous distribution and potential multifunctional cellular role. As optineurin plays a critical function across several key pathways, its dysfunction is likely to lead to the disruption of mechanisms that aim to maintain cell homoeostasis and thus contribute to the development of a number of human pathologies.

## Optineurin Protein Domain Structure and Interacting Partners

The human *OPTN* gene, containing three non-coding exons that makeup its 5′-untranslated region (UTR) and 13 exons that encode the 577 amino acid (66 kDa) protein, is ubiquitously expressed in most tissue and cell types (13). Four isoforms with identical open-reading frames have been reported to be generated through alternative splicing of the 5′-UTR (14).

**Figure 1:**
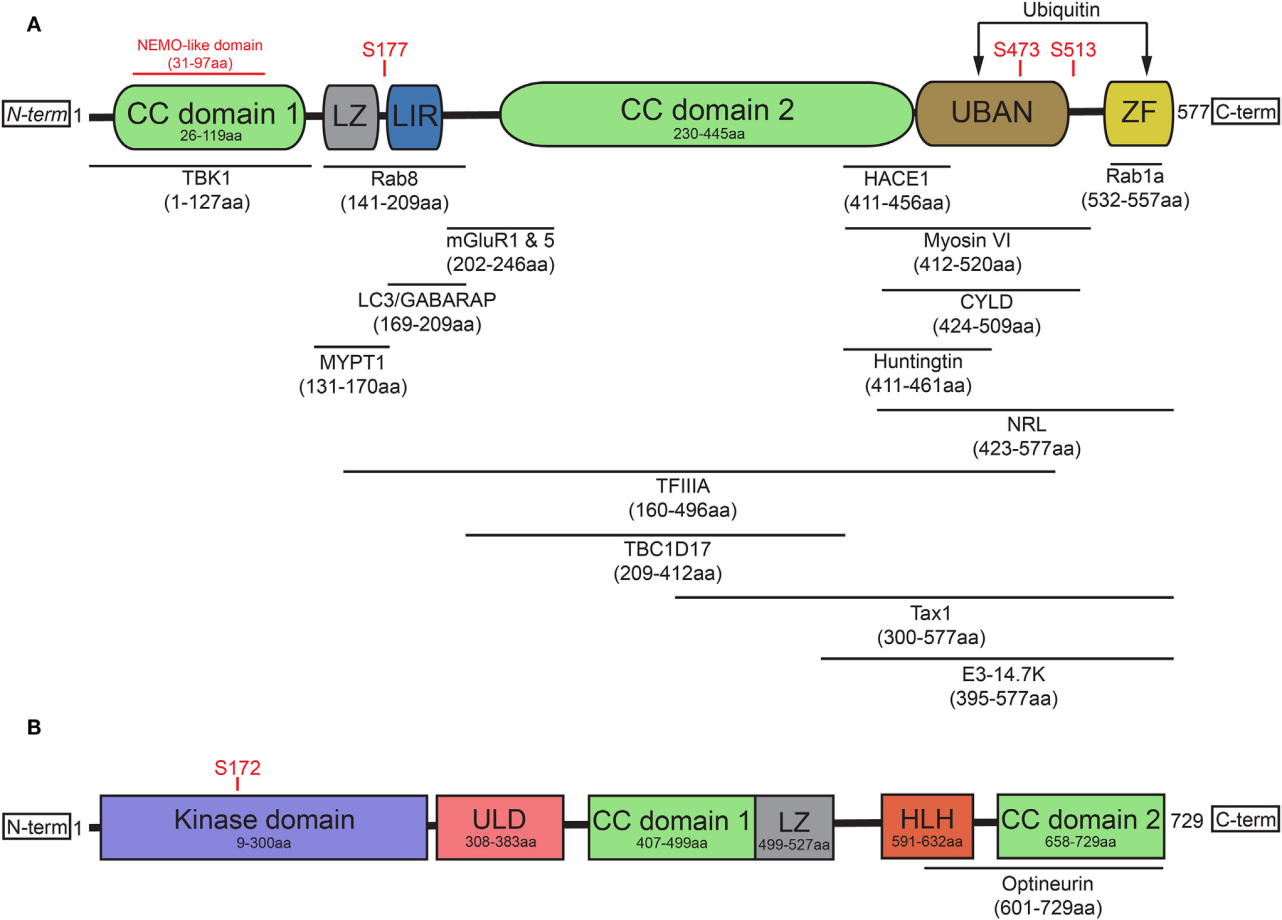
Optineurin and TBK1 both contain multiple structurally distinct domains associated with their regulation, binding, and activity. **(A)** Optineurin comprises two coiled-coil (CC) domains, a leucine zipper (LZ), an LC3-interacting region (LIR), UBAN domain, and a zinc finger (ZF) domain at it C-terminus. To date, a number of studies have identified the interacting regions of optineurin with its binding partners, defined in this figure. Serine phosphorylation sites are represented that regulate optineurin’s LC3-binding or ubiquitin-binding capacity. **(B)** TBK1 comprises a kinase domain, a ubiquitin-like domain (ULD), two CC domains, a LZ, and a helix-loop-helix (HLH) motif. Serine 172 represents the site that regulates TBK1’s kinase activity. TBK1 interacts with optineurin via its C-terminal HLH and CC domains.

*OPTN* originated from gene duplication of the NF-κB regulator NF-κB essential modulator (NEMO) (15) and contains two ubiquitin-binding motifs, which are the ubiquitin-binding domain (UBD) of ABIN proteins and NEMO (UBAN) domain and the zinc finger (ZF) domain (16). It has been previously suggested that the presence of two nearby ubiquitin-binding motifs within the protein may explain optineurin’s binding preference for longer polyubiquitin chains (17, 18). In addition to the aforementioned UBAN and ZF domains, optineurin also contains at least one leucine zipper (LZ), multiple coiled-coil (CC) domains, a NEMO-like domain, and a microtubule-associated protein 1 light chain 3 (LC3)-interacting region (LIR) (Figure 1A) (19).

The role of optineurin as an adaptor across many cellular processes is made possible by its ability to interact with a large number of proteins (Figure 1A). Through its functional interactions with TANK (TRAF family member-associated NF-κB activator)-binding kinase 1 (TBK1) (20–22), LC3 (22, 23), myosin VI (24–28), tax1 binding protein 1 (TAX1BP1) (29), Rab8 (25, 30), huntingtin (Htt) (30, 31), transferrin receptor (32), adenovirus E3-14.7K (1), receptor-interacting protein (RIP) (33), the bZIP transcription factor neural retina leucine zipper (34), myosin phosphatase targeting subunit 1 (35), transcription factor IIA (36), SOD1 (37), caspase 8 (38), HACE1 (39), CYLD (40), or metabotropic glutamate receptor 1 and 5 (41), optineurin can regulate a multitude of pathways. In addition to these interactions, optineurin can also oligomerise to form homo-hexameric structures (42), which are likely to have distinct roles from the monomeric form. The specific regulation, spatiotemporal dynamics, and cellular functions of many of these interactions will be discussed later in this review.

Post-translational modifications of optineurin also occur as part of its regulation. TBK1, a serine/threonine kinase, is one of the primary regulators of optineurin and mediates many of the optineurin-dependent cellular processes discussed in this review. To date, a number of disease-associated mutations, specifically in ALS and frontotemporal dementia (FTD), have been identified that perturb TBK1 binding with optineurin, resulting in dysfunction of trafficking pathways such as autophagy (43). Structurally, TBK1 contains an N-terminal kinase domain and ubiquitin-like domain (ULD), along with an α-helical scaffold dimerization domain (SDD) and adaptor binding (AB) domain within the C-terminal region (44, 45) (Figure 1B). Activation of TBK1 occurs through phosphorylation of the Ser172 residue within its kinase activation loop (46), inducing complete remodelling of this loop (47). Four dimerisation interfaces have been identified within TBK1, formed by the SDD interacting with either the N- or C-terminal lobes of the kinase domain, the ULD, or residues within the SDD itself (45). It may be the case that a dimeric form of TBK1 is maintained in an inactive state through prevention of Ser172 phosphorylation. Following specific stimuli, TBK1 is subsequently recruited to signalling scaffolds, whereby its clustering triggers the engagement of interdimeric interactions to promote Ser172 phosphorylation (47). Recruitment to discrete scaffolds, such as those that occur on the Golgi (48), or to polyubiquitylated optineurin, to regulate the interferon response (17, 49), may provide specificity in response to distinct stimuli, therefore allowing activation of specific pathways. TBK1 localisation is therefore critical in determining its activity and subsequently its impacts on optineurin function.

To date, less is understood about the spatiotemporal regulation of the TBK1/optineurin axis compared with the characterisation of their interactions. Indeed, TBK1 binding of optineurin, within the C-terminal CC domain (21) through polar and hydrophobic interactions (20), is required for TBK1-mediated phosphorylation of Ser177. This in turn has been shown to markedly enhance the LC3-binding capacity of the optineurin LIR (22, 50). Phosphorylation of optineurin by TBK1 at Ser473 and Ser513 also enhances its binding affinity for polyubiquitin chains via the UBAN domain (51, 52). These data demonstrate how the regulated dynamic binding capacity and post-translational modifications of optineurin are critical in modulating its function in cargo recognition during autophagy (Figure 1A). Throughout this review, we label optineurin as a receptor or an adaptor in accordance with either its function in cargo recognition within the lumen of the autophagosome or its ability to interact with cytosolic facing proteins on the external membrane of the autophagosome, respectively.

## Role of Optineurin in Signalling and Intracellular Trafficking

Optineurin is associated with a number of signalling pathways. In particular, it has been shown to play an important role in the regulation of signalling cascades critical to the innate immune response. Several studies have shown optineurin to act upstream of NF-κB, negatively regulating its activity. Interleukin-1 receptor-associated kinase 1, along with tumour necrosis factor (TNF) receptor-associated factor 6 (TRAF6), activates the innate immune response (53, 54) and is degraded in a proteasome-dependent manner upon its phosphorylation (55). Optineurin directly binds IRAK1 and prevents TRAF6 polyubiquitination, which is critical for its mediation of NF-κB activation (56). Optineurin also inhibits NF-κB activation through another C-terminal-dependent interaction with the deubiquitinase CYLD. This interaction mediates a subsequent interaction between CYLD and RIP (40), the latter acting as an adaptor upon its ubiquitination of NEMO, which senses the polyubiquitination of RIP and activates downstream NF-κB signalling via IκB kinase complex (57, 58). Optineurin directly competes with NEMO for the binding to ubiquitylated RIP (33) and recruits CYLD, which deubiquitinates RIP to inhibit NF-κB activation (40). Recently, it was shown that activation of T-cell receptor signalling triggers the degradation of optineurin to overcome optineurin’s negative regulation of NF-κB signalling, which acts to suppress T-cell activation (59). Interestingly, NF-κB upregulates *OPTN* expression (60), suggesting a negative feedback loop exists to ensure proper regulation of NF-κB signalling. Furthermore, optineurin inhibits the antiviral innate immune response by targetting CYLD to TBK1 to suppress its kinase activity, subsequently inhibiting interferon production (61).

Along with its regulation of signal propagation, optineurin also plays an essential role in the maintenance of organelle structure and function. Optineurin associates with the Golgi complex (62, 63) and through an interaction with the multifunctional actin motor protein myosin VI, functions to maintain the structural organisation of this organelle (26, 64, 65). Loss or mutation of optineurin in cell lines leads to Golgi fragmentation (26, 66–68) and although this was not replicated *in vivo* in zebrafish embryos (69), increased cell death and vesicle trafficking defects were observed. However, since the loss-of-function zebrafish model retains a low level of optineurin mRNA and possibly a truncated version of optineurin protein, it remains to be determined the extent of this phenotype (67). Alternatively, the role of optineurin in Golgi maintenance may therefore be cell type specific, or alternative/compensatory pathways may exist that can maintain Golgi morphology but do not necessarily rescue vesicular trafficking defects.

In addition, optineurin associates with Htt and Rab8 at the Golgi, where it acts as part of a complex to regulate post-Golgi trafficking of proteins (26), sorted by clathrin adaptor protein complex 1B and myosin VI (70). Mutations in Htt can uncouple the optineurin/Rab8 complex at the late Golgi compartment, resulting in decreased trafficking to lysosomes (71). Htt also functions as part of a number of vesicular trafficking pathways (72–74), which suggests that Htt defects may have wide-ranging impacts on optineurin function along related trafficking pathways. Rab8 is a critical component of the trafficking along the biosynthetic pathway from the trans-Golgi network (75, 76) and it also functions along other discrete endosomal routes. In particular, it has been shown that an optineurin interaction with the Rab-activating protein TBC1D17 regulates Rab8-dependent endosomal tubule formation and recycling of the transferrin receptor (77). Furthermore, optineurin is phosphorylated by Plk1 at Ser177, which dissociates optineurin from the Golgi through abrogation of a Rab8 interaction, facilitating its translocation into the nucleus to promote mitotic progression through regulation of Plk1 activity (35). Optineurin also functions post-Golgi to facilitate secretory vesicle fusion at the plasma membrane via an interaction with myosin VI (24). Therefore, optineurin may participate as a ‘keystone’ adaptor protein within these complexes to maintain Golgi organisation and coordinate multiple routes of post-Golgi trafficking.

Interestingly, it has also been shown that optineurin is required for the recruitment of ubiquitylated TBK1 to the Golgi apparatus, a critical step in TBK1 activation following viral RNA sensing as part of the innate immune response (48). Therefore, it is likely that optineurin association with the Golgi through its interaction with Rab8 (26) also recruits ubiquitylated TBK1 through its UBD (48), thus acting as a necessary precursor to the activation of this heterodimeric complex (20). The stabilisation of the TBK1/optineurin complex via ubiquitin could in turn allow for the enhanced propagation of optineurin-mediated signalling, as well as increasing its affinity for LC3 to promote autophagy progression, a mechanism we discuss in detail later in this review.

## Optineurin Regulation of Autophagy

The cellular mechanism to degrade cytosolic components is primarily carried out via the ubiquitin proteasome system (UPS) or autophagy. The latter process of autophagy, ‘cellular self-eating,’ acts to degrade proteins, organelles, and invading pathogens as part of a bulk process, whereas the UPS functions to degrade individual proteins (78). Indeed, both UPS and autophagic capacities are essential homeostatic pathways under basal conditions or in response to stress. Dysfunction in either is associated with the pathogenesis of a large number of disorders, ranging from neurodegenerative disease to cancer. Around 30% of newly synthesised proteins misfold (79), rendering them prone to aggregation. These aggregates cannot be efficiently degraded by the UPS, even resulting in inhibition of proteasomal functions (80, 81), and thus must be removed via autophagic mechanisms. It should, however, also be noted that significant cross-talk between the UPS and autophagy exists, despite the fact they are often considered as completely separate systems (82).

**Figure 2:**
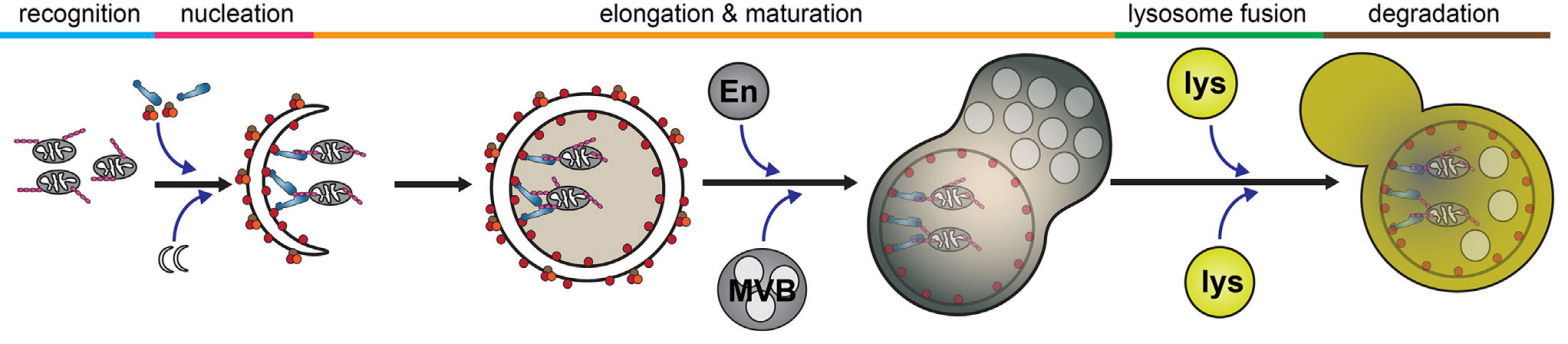
Selective autophagy. The autophagy pathway can be divided into five major steps: cargo recognition, phagophore nucleation, autophagosome elongation and maturation, fusion with the lysosome, and cargo degradation. Initial steps of cargo identification, as which occurs during mitochondrial capture, requires ubiquitination of a substrate and identification by autophagy receptors, such as optineurin, which facilitates the recruitment and nucleation of autophagosomal membrane to encapsulate the cargo. Subsequently, the autophagosome undergoes maturation following fusion with various endosomal vesicles and eventually fuses with the lysosome to facilitate cargo degradation. Abbreviations: En, endosome; MVB, multivesicular body; lys, lysosome.

Autophagy is a catabolic process by which intracellular components are engulfed and degraded. There are three forms of autophagy that can be differentiated by their function and mechanism of cargo delivery. These are chaperone-mediated autophagy, microautophagy, and macroautophagy. In this review, we will exclusively discuss the implications of macroautophagy, which requires the formation of a distinct organelle, the autophagosome. Although non-selective, bulk macroautophagy (herein termed autophagy) can occur under conditions of nutrient starvation to recycle cytosolic content, cargo-specific autophagy (termed selective autophagy) is critical in the removal of potentially cytotoxic components, such as damaged organelles, protein aggregates, and invading pathogens. This process can be divided into five basic stages: cargo recognition, autophagosome nucleation, autophagosome elongation and maturation, fusion with the lysosome, and degradation of cargo (Figure 2).

To correctly engage selective forms of autophagy to mediate the degradation of specific substrates, autophagy receptor proteins such as optineurin, nuclear dot protein 52 (NDP52), TAX1BP1, neighbour of BRCA1 gene 1 (NBR1), or p62 are required (22, 28, 83–85). Substrates to be degraded are ubiquitylated and recognised by UBDs, specific for certain ubiquitin linkages types, present within the autophagy receptors. Through an additional LC3 interacting region (LIR), these receptors can directly interact with autophagosomal membrane, thus facilitating cargo recognition, trafficking, and degradation (86).

To date, over 30 autophagy-related (*ATG*) genes have been identified in the yeast, *Saccharomyces cerevisiae* (87, 88). In mammals, these have been shown to be involved in both ubiquitin-dependent and -independent mechanisms of autophagy (89). In yeast, Atg8, an ubiquitin-like protein, conjugates to phosphatidylethanolamine to be inserted into lipid membranes to mediate tethering and formation of the autophagosomal double membrane (90–92). The mammalian Atg8 homologues, LC3, γ-aminobutyric-acid-type-A-receptor-associated protein (GABARAP), and Golgi-associated ATPase enhancer of 16 kDa, were then later identified to undergo post-translational modifications to form species that can associate with autophagosomal membranes (93–96). p62 was subsequently shown to directly bind, via a LIR, to both LC3 and GABARAP (97) and ubiquitin-labelled proteins via its ubiquitin-associated (UBA) domain (98). Importantly, formation and clearance of ubiquitin-positive protein inclusions is ablated in p62-deficient cells (97, 99). Thus, p62 acts as a receptor protein between ubiquitylated protein aggregates and the LC3-positive autophagosomal membranes.

Similar to p62, other autophagy adaptors such as optineurin, TAX1BP1, NDP52, and NBR1 also directly bind ubiquitin and LC3 to coordinate the autophagosome-mediated engulfment of cargo. In particular, optineurin was first identified as an autophagic receptor through its interaction with Atg8-related proteins in a yeast two-hybrid assay and its localisation to LC3-positive autophagosomal membranes upon induction of xenophagy, the selective autophagy pathway for pathogens (22). Here, the authors identified that optineurin interacts with LC3 and GABARAP through an LIR located between its CC domains. Crucially, the demonstration that phosphorylation upstream of the optineurin LIR regulates its interaction with LC3, and thus its autophagic function, was a novel finding at the time showing a further level of regulation for autophagy receptors. In addition, optineurin’s ability to function as an autophagy receptor has relevance to distinct pathological mechanisms, as it was recently shown to directly interact with the endoplasmic reticulum stress protein IRE1α and function to suppress activation of the unfolded protein response via mediating the autophagic degradation of IRE1α (100).

The ‘ubiquitin code,’ which regulates signal transduction and degradation of labelled substrates, has an inherent complexity. This is due to the occurrence of both mono- and poly-ubiquitin chain types as well as the multiple layers of lysine-dependent heterotypic polyubiquitin chain linkages, such as those mediated by K6, K11, K27, K29, K33, K48, or K63 (101). Broadly, there are two routes of degradation for ubiquitylated substrates; UPS- or autophagic-mediated degradation. K63-linked polyubiquitin chains are thought to primarily determine autophagic clearance of a substrate (84, 102, 103). Optineurin contains two UBDs, an UBAN domain and ZF domain. The UBAN and ZF domains bind K63- but not K48-linked polyubiquitin chains (15, 16, 33) suggesting optineurin primarily functions along the autophagic degradation pathway or alternatively regulates signal propagation, as which occurs along the NF-κB pathway. However, optineurin, TAX1BP1, and NDP52 preferentially bind different types of ubiquitin chains (15), which may be critical in determining their cargo specificity during autophagy.

**Figure 3:**
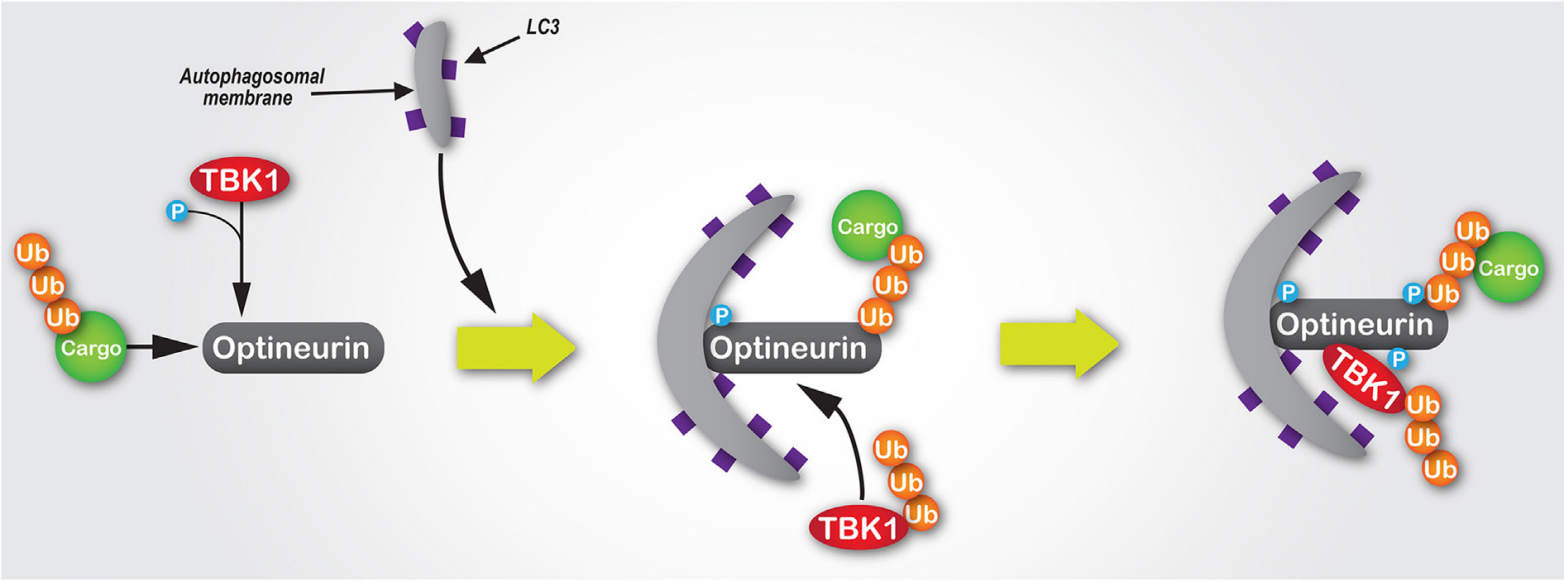
The mechanisms of the TBK1/optineurin complex during autophagy. Optineurin interacts with ubiquitylated cargo via its UBAN and zinc finger domains. TBK1 is then recruited via an interaction with optineurin to facilitate its phosphorylation at Ser177, which enhances its light chain 3 (LC3)-binding capacity and recruitment of autophagosomal membrane. Subsequently, TBK1-mediated phosphorylation of optineurin at Ser473 and Ser513 enhances its polyubiquitin-binding capacity, thus stabilising its interaction with ubiquitin-labelled cargo. Since K63-linked polyubiquitylation of TBK1 is required for its activation, as well as its recognition and recruitment by Golgi-localised optineurin, we would hypothesise that during autophagosome formation ubiquitylated TBK1 is recruited by optineurin, where it is activated and in turn phosphorylates optineurin, thus creating a positive signal amplification loop through the recruitment and stabilisation of the TBK1/optineurin heterodimeric complex on ubiquitylated cargo.

Intracellular pathogens, such as *Salmonella enterica*, which escape into the cytosol from a vacuolar compartment are targetted and degraded by the autophagy machinery (104). The capacity of optineurin to function as an autophagy receptor, which is enhanced by Ser177 phosphorylation, is critical to suppress the hyperproliferation of cytosolic *S. enterica* (22). TBK1, a critical regulator of autophagy (105), binds to optineurin (21) and induces phosphorylation within the N-terminal LC3-interacting motif of optineurin (22) (Figure 3). A similar axis has also been observed with TBK1-dependent modulation of NDP52 function, which promotes autophagy of *S. enterica* (85), suggesting the potential for synergism or functional redundancy between autophagy receptors in the innate immune response. Some suggestion of this has already been observed, whereby multiple receptors function cooperatively along the same pathway (22, 106). However, it has also been shown that for both xenophagy and the selective mitochondrial pathway, mitophagy, optineurin, and p62 are independently recruited to separate autophagosomal subdomains (22, 23), suggesting they function along parallel pathways to facilitate pathogen and mitochondrial degradation.

In the case of *Listeria monocytogenes*, upregulation of optineurin occurs in response to the bacterial expression of listeriolysin O (LLO) (107), a pore-forming cytolysin that allows the bacteria to escape from a vacuolar compartment into the cytosol following host entry (108). Here, TBK1 activity enhances optineurin-mediated clearance of the pathogen, whilst a reduction in optineurin expression results in less autophagosomal clearance of *L. monocytogenes* (107). These data together are indicative of the importance of the TBK1–optineurin axis in the clearance of several pathogenic bacteria. It also suggests that under these conditions, this optineurin-regulated immune defence system has specifically evolved to detect the LLO-mediated translocation of bacteria into the cytoplasm.

Further highlighting the importance of the TBK1–optineurin axis, pharmacological inhibition of TBK1 activation using BX795 (109) inhibits optineurin phosphorylation and subsequent LC3 recruitment (22). Moreover, activation of the TBK1–optineurin complex in mouse bone marrow-derived macrophages is perturbed by the ubiquitin-binding defective *OPTN*^D477N^ mutant (17, 110), suggesting that the binding of ubiquitin-tagged cargo by optineurin is a necessary precursor to its phosphorylation, and thus activation of this complex. Interestingly, TBK1-mediated phosphorylation of optineurin’s UBAN domain at S473 further enhances optineurin’s capacity to bind ubiquitin chains (52) (Figure 3). Indeed, optineurin has also been shown to directly regulate TBK1 activity (48). K63-linked polyubiquitination of TBK1 at residues K30 and K401 is required for TBK1 activation (111). These ubiquitin chains are sensed by optineurin localised at the Golgi apparatus via its interaction with Rab8 (26), which results in the formation of a complex between optineurin and TBK1, with the latter activated by trans-autophosphorylation (48).

Optineurin is also a key adaptor protein for the actin motor protein myosin VI (112). This interaction is critical for the spatiotemporal regulation of many optineurin-mediated functions, including autophagy and secretory vesicle fusion (24, 27, 28). There are around 40 different myosins expressed in humans (113) and due to the association of myosin dysfunction in a number of diseases, the development of small molecules to manipulate their function is a growing area of investigation (24). However, unlike other myosins, myosin VI movement is towards the pointed (minus) ends of actin filaments (114) using large powerstroke movements achieved through significant conformational rearrangement (115–117). Whilst the N-terminal motor domain, conserved across myosins, undergoes adenosine triphosphate (ATP)-dependent conformational changes to induce motor translocation (118), the C-terminal tail region is divergent across the myosin family and thus confers cargo specificity via direct interactions (112). Upon the binding of cargo, for example, via optineurin as an adaptor, myosin VI is able to dimerise and potentially function as a processive motor (119). To date, multiple binding motifs within the tail region of myosin VI have been identified, which allow specific interactions with a range of proteins that function in membrane trafficking (26, 28, 120–127). In particular, the RRL motif within the myosin VI tail is required for its interaction with optineurin, as well as the other autophagy receptors TAX1BP1 and NDP52 (26, 126, 127).

Mutation within, or deletion of, the optineurin UBD perturbs optineurin pull down of myosin VI, as well as target of myb protein 1 (Tom1) (128), highlighting the importance of this region in the interaction with the myosin cargo-binding tail and its potential to facilitate larger scale adaptor protein complexes. Recent data have shed further light on this. Within the C-terminal region of myosin VI, a motif interacting with ubiquitin domain exists (129). A second region, encompassing the RRL motif, was subsequently identified and termed the myosin VI ubiquitin-binding domain (MyUb) (123). Here, the authors found that residues R1117 (part of the RRL motif) and I1104 within the MyUb domain are critical for MyUb structural integrity and the binding of ubiquitin conjugated to optineurin, respectively. This may suggest that optineurin, separate to its function as a cargo-binding receptor that binds ubiquitin upon phosphorylation by TBK1, may act as an adaptor protein by interacting with the myosin VI MyUb domain or RRL motif to facilitate autophagosomal maturation.

The optineurin–myosin VI complex likely regulates a key aspect of autophagy, which is to facilitate the maturation of the autophagosome and its fusion with the lysosome (28, 130). In particular, myosin VI, through a direct interaction with optineurin via its RRL motif (26), delivers Tom1-positive endosomal membranes to autophagosomes, which is required for autophagosome–lysosome fusion (28). This holds significance because the origins of the autophagosomal membrane are wide-ranging and highly debated within the literature, with recruitment coming from the ER (131, 132), endosomal compartments (133–136), plasma membrane (137), mitochondria (138), and Golgi (139, 140) all contributing to nucleation and elongation of the phagophore membrane. It has also been demonstrated that autophagosomal membranes derive from ER–mitochondrial contact sites (141), as well as the ER–Golgi intermediate compartment (26, 142, 143). Tom1 is an alternative endosomal sorting complex required for transport (ESCRT) class 0 protein (144), a family of trafficking proteins required for cargo sorting along the endocytic route and in the autophagy pathway (145), and binds the WWY motif of myosin VI, unlike optineurin, NDP52, and TAX1BP1 which bind the RRL motif (28). Although multiple studies had previously shown Tom1 and myosin VI to interact (122), the more recent observations discussed here (28, 123, 129) may suggest how this specific and dynamic pathway is tightly regulated.

The capacity of optineurin to bind both ubiquitylated cargoes and autophagosomal LC3 via its UBD and LIR, respectively (19), and myosin VI in an ubiquitin-dependent (123) or -independent manner may represent distinct autophagic steps. In this paradigm, it may be that a specific stimulus results in TBK1 recruitment and subsequent phosphorylation of optineurin at sites of cargo recognition and autophagosome formation to enhance its binding to LC3 (22) and ubiquitin (52). Separately, the conjugation of cytosolic optineurin to ubiquitin may enhance its interaction with myosin VI, via the MyUb RRL motif (123), to recruit it to LC3-positive membranes and form an adaptor/membrane/motor complex to promote autophagosomal maturation. It is therefore important to note that optineurin likely has a dual function during autophagy, functioning as a cargo receptor in the lumen of the autophagosome and also functioning as an adaptor protein on the cytosolic face of the autophagosome. More recent data further implicate optineurin in autophagosomal maturation in neurons through an interaction with the GTPase Rab1a (146). Optineurin also mediates the recruitment of the Atg12-5-16L1 complex to promote autophagosomal elongation (147), suggesting a role distinct from its cargo-binding capacity. In addition, other autophagy receptors could play a cooperative role alongside optineurin. For example, NDP52 recruitment of TBK1 to autophagosomes via the formation of an ubiquitin-sensing complex with Nap1 and Sintbad (85) could stimulate the formation and stabilisation of the heterodimeric TBK1–optineurin axis. Moreover, optineurin, TAX1BP1, and NDP52 preferentially bind different types of ubiquitin chains (15), which may be critical in regulating their cargo specificity. Interestingly, the optineurin paralogue NEMO is negatively regulated by the E3 ubiquitin ligase TRIM29 via interactions within its CC domain, resulting in the ubiquitylation and degradation of NEMO (15, 148). Whether a similar mechanism exists to regulate optineurin function remains to be determined, but this may indicate the existence of a further mode of optineurin regulation.

## Optineurin Function During Mitophagy

Mitochondria are a critical organelle in the eukaryotic cell, with most cellular ATP produced by oxidative phosphorylation (OXPHOS) within the mitochondrial matrix. Mitochondria provide the major source of intracellular cytotoxic reactive oxygen species (ROS) (149) as a by-product of OXPHOS, with ROS production increasing upon mitochondrial damage. It is therefore crucial that the accumulation of dysfunctional mitochondria is effectively prevented through homeostatic mitochondria quality control pathways, such as mitophagy. Failure of these mechanisms is strongly associated with a number of age-related diseases, such as Parkinson’s disease (150). Mitophagy, a term originally coined over a decade ago (151), is the selective autophagic removal of damaged mitochondria within a cell, although the UPS is also a critical component of this pathway (152–155). More recently, the role of receptors/adaptors such as optineurin in mitophagy has begun to emerge, which has resulted in their investigation in greater detail.

The most well studied form of mitophagy is regulated by the PTEN-induced putative kinase 1 (PINK1)/Parkin axis, although alternative pathways have been shown to exist. Under ‘normal’ or ‘healthy’ conditions, PINK1 is rapidly imported into mitochondria via TOM40 and translocation of inner membrane pores (156) in a mitochondrial membrane potential-dependent manner (157–159). Following its import, PINK1 undergoes intermembrane degradation by mitochondrial processing peptidase and presenilin-associated rhomboid-like protein (160–162), with the residual N-terminus then being exported into the cytosol for proteasomal turnover (163).

Upon mitochondrial damage, PINK1 is stabilised and selectively accumulates on the mitochondrial outer membrane (MOM), where it recruits and activates Parkin (158, 159, 164). PINK1 is critical for a number of post-translational modifications to Parkin (165, 166), MOM proteins (167), and ubiquitin (168–171), as well as promoting fission to isolate damaged mitochondria for degradation (172). Parkin subsequently ubiquitylates a number of MOM proteins (173–175), in addition to RHOT1/2 (Miro in *Drosophila*), a small GTPase involved in mitochondrial transport, resulting in the arrest of mitochondrial trafficking (176).

For mitophagy to correctly function and damaged mitochondria to be selectively degraded, autophagy receptors once again represent critical components of the pathway. The mitochondrial protein Nix has been identified as an autophagy receptor for the targetted clearance of mitochondria (177), which is regulated by its phosphorylation (178). Although p62 has been shown to act as a receptor during mitophagy (174), its importance has since been disputed (23, 179). We would suggest that both functional redundancy and cooperativity are likely to exist between autophagy receptors with respect to their role during mitophagy. It may be the case that specific receptors are critical at distinct points during mitophagy, or that they only function under different types of mitochondrial stress and in certain cell lines. For example, mitochondrial damage induced by oxidative stress may result in the activation of a different mitophagy pathway compared with pharmacological uncoupling of membrane potential. Although p62 is recruited to uncoupled mitochondria in HeLa cells (179), optineurin is also recruited under the same conditions (23) where it induces autophagosome assembly (180).

Optineurin, along with NDP52, is recruited by PINK1 to damaged mitochondria, but in a Parkin-independent manner (181). Optineurin then preferentially binds linear ubiquitin chains via its UBAN domain (38), with TBK1 activity regulating this interaction by phosphorylation of residues within this domain (51). Although the phosphorylation of ubiquitin has been suggested to be critical in PINK1/Parkin-dependent mitophagy (170) and TBK1-mediated phosphorylation of optineurin on Ser473 facilitates its binding of pSer65 ubiquitin chains on mitochondria (52), conflicting reports have also emerged on whether optineurin activity requires ubiquitin phosphorylation in the context of mitophagy (51, 181, 182). It may be the case that these phosphorylation events are dispensable for mitophagy under certain conditions, but not others.

The fact that p62 and optineurin are recruited to distinct domains on damaged mitochondria to facilitate the separate roles of mitochondrial aggregation and LC3 recruitment, respectively (23), demonstrates the functional divergence of autophagy receptors. More recently, the divergent pathways of the overall process of mitophagy have also become better understood. Degradation of mitochondrial proteins can occur via a pathway in which mitochondria-derived vesicles (MDVs) bud off from the organelle (183–185) in a Parkin/PINK1-dependent manner (186), with Syntaxin-17 mediating MDV fusion with endolysosomal compartments (187). This pathway is likely to represent both normal physiological recycling of mitochondrial proteins and the disposal of mitochondrial components damaged by low level stress. Although no direct assessments have been made to date, we would hypothesise that proteins such as optineurin may act as receptors and/or adaptors in this lysosomal degradation pathway as the loss of Parkin ubiquitin ligase activity perturbs the MDV pathway (186), suggesting that receptors with ubiquitin-binding capacity may be required downstream of Parkin to facilitate degradation. In addition, trafficking of MDVs containing mitochondrial proteins to lysosomes is likely to require adaptor protein interactions with molecular motors such as myosin VI to facilitate cargo delivery.

Such alternate mitophagic pathways could be activated only under specific stress conditions, whereby distinct autophagy receptors undergo mitochondrial recruitment. Some evidence of this has already been observed, whereby the receptor TAX1BP1 interacts with Parkin upon mitochondrial uncoupling, but only when fusion events are also inhibited by Bafilomycin A1 (175). This could suggest that specific autophagy receptors only play a role in this pathway if other stress conditions occur in parallel, or alternatively illustrate that these interactions are transient and the inhibition of other pathways leads to their retention. Indeed, this may even better represent actual physiological disease conditions, where cells are likely to be undergoing multiple stresses whilst trying to maintain homoeostasis. Extensive further work is therefore needed to delineate the specific role of receptors, such as optineurin, during mitophagy using physiologically relevant disease models.

## Optineurin in Human Disease

### Primary Open-Angle Glaucoma

As previously discussed, optineurin has been associated with a number of diseases across a wide range of genetic and functional-based studies. The first proven association with disease was over a decade ago when mutations in *OPTN* were shown to cause an autosomal dominant form of hereditary glaucoma (2). Here, the initial studies suggested that optineurin plays a neuroprotective role, a hypothesis that has been supported by numerous subsequent publications (188–191).

Glaucoma is a disease characterised by the progressive degeneration of the optic nerve. This optic neuropathy is the primary cause of irreversible blindness worldwide, with POAG being the most common subtype (192). Although often classed as a neurodegenerative disease, it has been hypothesised that it is a primary optic neuropathy with secondary pathogenic effects in the central nervous system (193). The bilateral blindness that results from glaucoma is a result of the progressive loss of retinal ganglion cells (RGCs) in the optic nerve head (194). A number of studies have suggested that mutations in optineurin that cause glaucoma are a result of defective autophagy (195). Furthermore, this pathology resulting from autophagic defects may be limited specifically to dysfunction in optineurin-mediated autophagy as a small-scale genetic study did not find mutations in the *SQSTM1* gene encoding the autophagy receptor p62, also phosphorylated by TBK1 (105), in patients with NTG (196).

The optineurin E50K mutation, a primary cause of POAG-induced blindness (2), impairs autophagy. Indeed, in this initial study by Rezaie et al. to identify *OPTN* mutations as causative of glaucoma, the E50K mutation segregated with the NTG phenotype within a large family, providing solid evidence for their hypothesis and was associated with 16.7% of the familial NTG cases investigated. The extension of these data into E50K transgenic mouse models has further supported this hypothesis (190, 197, 198), with mice specifically exhibiting pathological features of POAG when physiological relevant levels of the transgene were expressed (199). Cell death is also induced in mouse photoreceptor cells derived from retinal tumours expressing either E50K or M98K glaucoma-associated variants (200) (Table 1).

**Table 1:** Identified and characterised optineurin mutants associated with POAG and ALS.

Mutation	Disease	Functional impacts	Interactions	Reference
E50K	POAG	Autophagy dysfunction; photoreceptor cell death; altered mitochondrial dynamics; increased ROS; mitochondrial loss; increased expression of Bax	Enhanced TBK1 interaction; disrupted Rab8 interaction; enhanced oligomeric state of optineurin	(2, 36, 190, 200–202)

M98K	POAG	Photoreceptor and RGC cell death; increased degradation of TfR; enhanced S177 phosphorylation; increased autophagic cell death	Enhanced Rab12 interaction; enhanced binding to TBK1	(200, 203, 204)

H486R	POAG, JOAG	NF-κB dysregulation	Disrupted CYLD interaction; decreased ubiquitin binding	(40, 205, 206)

E478G	ALS	Lack of mitochondrial translocation; cytoplasmic inclusions; NF-κB dysregulation	Interaction with SOD1 aggregates intact; lack of ubiquitin binding	(8, 18, 37, 181, 207)

D398X (truncation)	ALS	Lack of mitochondrial translocation; NF-κB dysregulation; Golgi fragmentation	Lack of ubiquitin binding	(8, 181, 207)

R96L	ALS	Golgi fragmentation; predicted gain-of-function	Enhanced Rab8 binding	(207)

Q165X (truncation)	ALS	Predicted loss-of-function	Predicted disruption of Rab8, myosin VI, Htt, and ubiquitin binding	(10)

Q454E	ALS	Reduced NF-κB inhibition	Unknown	(10, 38)

*Identified optineurin disease mutants in POAG, JOAG, and ALS. In addition, the functional impacts and the effects on protein–protein interactions of these mutants are described*.

*TfR, transferrin receptor; ROS, reactive oxygen species; NF-κB, nuclear factor kappa B; RGC, retinal ganglion cell; TBK1, tank-binding kinase 1; CYLD, cylindromatosis lysine 63 deubiquitinase; Htt, huntingtin; SOD1, superoxide dismutase 1; POAG, primary open-angle glaucoma; JOAG, juvenile open-angle glaucoma; ALS, amyotrophic lateral sclerosis*.

At the subcellular level, the E50K mutation enhances its interaction with TBK1 (21), which disrupted proper oligomerisation resulting in its insolubility (201). This E50K mutation has also been shown to perturb optineurin’s interaction with Rab8 (77, 190, 208), a critical regulator of vesicular trafficking. The M98K mutation, found in 13.6% of NTG cases in one study (2), enhances the interaction of optineurin with Rab12 (203), a GTPase involved in vesicular trafficking and lysosomal degradation of the transferrin receptor (209). This enhanced interaction lead to the increased degradation of the transferrin receptor and RGC death (203). Furthermore, M98K demonstrates enhanced binding to TBK1, which in turn leads to enhanced Ser177 phosphorylation and thus optineurin activation in a TBK1-dependent manner, resulting in activation of autophagic cell death (204). In neuronal RGCs, the overexpression of wild-type or E50K optineurin compromises UPS-mediated turnover of optineurin leading to the accumulation of autophagosomes and apoptosis (202). It would appear that cells must maintain functional levels of optineurin and that the alteration of this homeostatic balance results in autophagic-induced cell death and/or autophagic dysfunction (Table 1).

The aberration of mitochondrial homoeostasis is also associated with glaucoma (210, 211). In transgenic mice or *in vitro* cultured RGCs, E50K expression alters mitochondrial dynamics and promotes expression of the proapoptotic protein Bax, leading to retinal cell death. In addition, this mutation resulted in mitochondrial loss through the induction of mitochondrial fission and the formation of mitochondrial-containing autophagosomes, as well as increased ROS production (212). This dysfunction in mitochondrial regulation may elucidate why oxidative stress-induced retinal cell death is associated with this particular optineurin mutation (189, 213).

### Amyotrophic Lateral Sclerosis

Amyotrophic lateral sclerosis is a neurodegenerative disease associated with mitochondrial dysfunction with respect to their function, morphology, transport, and turnover (214–217). Mutations in optineurin have been identified in ALS patients, as well as mutations in its complex binding partners TBK1 and p62, suggesting that autophagic dysfunction is the common pathway (8, 10, 18, 207). In support of this, optineurin and TBK1 mutations perturb the recruitment of LC3-positive membrane to damaged mitochondria, leading to less efficient mitophagy (50), which could account for some cases of mitochondrial dysfunction observed in ALS.

Disruption of the TBK1–optineurin interaction and their co-dependent regulatory mechanisms can be attributed to disease pathology. For example, whereas the glaucoma-associated E50K mutation in optineurin enhances its interaction with TBK1 resulting in impacts on the oligomeric state of optineurin, the ALS-associated E696K mutation of TBK1 abolishes its interaction with optineurin leading to a failure of mitochondrial translocation (20, 52). In addition, optineurin may be activated by TBK1-mediated Ser177 phosphorylation to induce autophagic clearance of protein aggregates in an ubiquitin-independent manner via its C-terminal CC domain. Interestingly, in this study, the optineurin UBAN mutant E478G still interacted with SOD1 protein aggregates, whereas depletion of optineurin in this ALS zebrafish model resulted in motor axonopathy (37). Importantly, these data have implications for both ALS and Huntington’s disease. Furthermore, mutations in *TBK1* have more recently been associated with the development of FTD associated with ALS (218–221). *SQSTM1* mutations in FTD and FTD with ALS have also been identified (222), which would indicate that autophagic dysfunction is at the heart of these diseases. It may therefore be the case that some TBK1 mutation-associated phenotypes in FTD/ALS occur through an optineurin-mediated action with resulting autophagic defects driving the degenerative pathology.

Many of the optineurin mutations associated with ALS are located within the UBAN domain, thus disrupting ubiquitin binding (8). ALS-associated optineurin mutations E478G and Q398X (both within the UBAN domain), as well as the ubiquitin-binding deficient D474N, do not translocate to mitochondria (181) (Table 1). However, the authors did find that the expression of the glaucoma-associated E50K mutation and the phospho-deficient S177A could marginally rescue mitophagy. This limited rescue may be explained by the fact that E50K and S177A optineurin mutants, unlike ubiquitin-binding deficient mutants, are still recruited to damaged mitochondria where they are still able to exhibit some activity, resulting in very low level recruitment of TBK1. These data are therefore indicative of an optineurin-mediated system in which its interaction with ubiquitin is most critical for mitophagy. A current hypothesis is therefore that mutations disrupting the ubiquitin-binding capacity of optineurin prevents efficient mitophagy in neurons and leads to the accumulation of cytotoxic dysfunctional mitochondria (180). p62 and optineurin are recruited to discrete domains on damaged mitochondria (23), suggesting distinct functional mechanisms exist. However, it would appear that disrupting just optineurin activity alone is enough to induce ALS pathology. The reason why neurodegeneration only occurs in specific neuronal subtypes carrying these ALS-associated familial mutations is likely due to these cells unique energetic demands and susceptibility to mitochondrial damage alongside their limited capacity for mitochondrial homeostatic pathways. Nevertheless, as wild-type optineurin binds and inactivates caspase-8 (38), mutations that result in a loss of this activity may potentiate the apoptotic pathway that occurs in ALS-associated pathologies following optineurin dysfunction.

### Other Diseases

In addition to ALS and FTD, the TBK1/optineurin axis may also be implicated in the pathogenesis of other neurodegenerative disorders. Indeed, a patient carrying the optineurin E478G mutant was clinically diagnosed with both ALS and Parkinson’s disease, with autopsy analysis showing degeneration of the substantia nigra, as well as the presence of tau-positive neurofibrillary tangles and α-synuclein-positive Lewy bodies (223). As optineurin acts as a receptor during mitophagy (23), a pathway in which its dysfunction is known to cause Parkinson’s (224), it is possible that hereditary or somatic mutations in genes encoding optineurin or TBK1 may lead to a Parkinsonian progression through mitophagic perturbation.

Trinucleotide expansions within the *HD* gene encoding the Htt protein result in the progression of the devastating neurodegenerative disorder Huntington’s disease (225, 226). Due to its interaction with Htt (30), a protein known to regulate a number of vesicular trafficking pathways (71, 227, 228), optineurin is of significant interest in Huntington’s research. Although optineurin interacts with Rab8 and Htt at the Golgi (26), a localisation that is disrupted by mutant Htt resulting in lysosomal impairment (71), and is found in Htt protein inclusions observed in the cortex of Huntington’s patients (229), it is currently not known what role optineurin plays in the progression of the disease. Nevertheless, because optineurin is involved in the autophagic clearance of protein aggregates (37) and its abundance/neuronal distribution may confer susceptibility to Htt inclusions (230), its role in mediating clearance pathways may offer novel therapeutic targets as our understanding grows.

The impairment of vesicular trafficking and autophagy is not just associated with neurodegeneration, but has also been linked to a number of cancers (231–233). HACE1, an E3 ubiquitin ligase and potent tumour suppressor (234), ubiquitylates optineurin which promotes its interaction with p62 and induces autophagy (39). This accelerated degradation lead to a suppression of ROS and reduction of tumourigenicity of human lung cancer cells. Thus, optineurin-induced autophagy appears to represent a potential tumour suppressing pathway in some cancers.

## Conclusion

Both autophagy and mitophagy have been implicated in cell survival and death pathways by a number of studies. The role of optineurin in these pathways currently remains relatively unexplored. Dysfunction in autophagy and mitophagy is associated with a number of neurodegenerative diseases and so questions therefore remain as to how optineurin-mediated autophagy, and its dysfunction, plays a role in directing neuronal death pathways under specific stress conditions. As multiple distinct pathways exist within each form of selective autophagy, which involves a number of distinct autophagy receptor and adaptor proteins, our understanding of which of these proteins play a role across each discrete pathway must be improved. For example, it is clear that the TBK1–optineurin complex plays a pivotal role during the innate immune response to target unwanted cellular pathogens, but how it spatially and temporally regulates this process with respect to related autophagy receptors has not yet been clearly defined. In addition, the outcome of TBK1 kinase activity may be regulated by the level and duration of activation, as well as by cross-talk between other kinase classes (235). Therefore, TBK1 regulation of autophagy may also occur in this manner, whereby only specific levels or discrete localisation of TBK1 activity leads to the activation of optineurin-dependent autophagy, thus allowing the cell to distinguish between different stimuli and mount the appropriate autophagic response. Nevertheless, disease-causing mutations in optineurin that result in the presentation of autophagic defects in patients highlights the central role that is played by this protein in the regulation of these cargo-specific membrane trafficking and recycling pathways.

## Author Contributions

TR performed the literature research and wrote the manuscript. DT coordinated the study, performed the literature research, and edited the manuscript.

## Conflict of Interest Statement

The authors declare that the research was conducted in the absence of any commercial or financial relationships that could be construed as a potential conflict of interest.
